# How Wide the Divide? – Theorizing ‘Constructions’ in Generative and Usage-Based Frameworks

**DOI:** 10.3389/fpsyg.2021.601303

**Published:** 2021-02-26

**Authors:** Matthew T. Carlson, Antonio Fábregas, Michael T. Putnam

**Affiliations:** ^1^Department of Spanish, Italian, and Portuguese, Program in Linguistics, Center for Language Science, The Pennsylvania State University, University Park, PA, United States; ^2^Department of Language and Culture, University of Tromsø, Tromsø, Norway; ^3^Department of Germanic and Slavic Languages, Program in Linguistics, Center for Language Science, The Pennsylvania State University, University Park, PA, United States

**Keywords:** constructions, Minimalism, emergence, exoskeletal, Nanosyntax, Construction Grammar

## Abstract

What is the nature and function of mental representations in cognitive science, and in human language in particular? How do they come into existence and interact, and how is the information attributed to them stored in and retrieved from the human mind? Some theories treat constructions as primitive entities used for structure-building, central in both production and comprehension, while other theories only admit construction-like entities as devices to map the structure into semantics or to relate them to specific morphophonological exponents. In this positional piece, we seek to elucidate areas of commonality across what have traditionally been divergent approaches to the role of constructions in language. Here we outline a robust specification of the differences in how chunks of structure containing information are treated in the two main approaches, and we seek to offer a path toward a more unified theoretical stance.

## Introduction

Irrespective of the various traditions scholars primarily associate themselves with (e.g., cognitive science, linguistics, psychology, etc.), as researchers interested in cognition and the study of the structural properties of human language, we are forced to come to terms with defining the frequent and systematic properties that constitute its fundamental building blocks. To put it bluntly, we are still collectively searching for and theorizing about the most appropriate, economical, and effective ways to describe these mental representations, be they single items or objects that are themselves non-trivially composed (let’s call these elements constructions), or atomic units and the primitive operations by which they give rise to composed units.

We begin by opposing theories in which mental representations are built, by hypothesis, from single units and the notion of construction is rejected as an ontological component, with theories that take constructions to be fundamental, eschewing the idea of smaller basic units except insofar as these emerge from correspondences among the constructions themselves. The mission of identifying these internally composed building blocks is far bolder than achieving mere descriptive adequacy in identifying the levels of representation of the human mind and their properties. The bigger challenge, as expressed by Chomsky (2005); Christiansen and Chater (2016), and others, is to extend beyond mere descriptive adequacy and explore how our ideas about constructions connect with other aspects of our biological endowment, the ontogenetic and phylogenetic development of language, and the socio-cultural environment that also have made lasting marks on the structural design of language in our species. We are thus called to advance a theory of constructions and the human language faculty (whether or not this is held to include domain-specific properties of the mind-brain) that seeks to achieve explanatory adequacy. This positional essay is an attempt to draw light on the common properties and existing fundamental differences of two competing theoretical models and their treatment of constructions; namely, (1) Construction Grammar (CxG, which in turn stands for a family of theories that have been developed in different versions by Kay and Fillmore (1999), Goldberg(1995; 2006; 2019), and Michaelis (2012); cf. also Fillmore and Kay (1993); Croft (2001) radical construction grammar, Boas and Sag (2012) sign-based construction grammar and some versions of cognitive grammar, such as Langacker (1987) and (2) Exoskeletal variants of the Minimalist Program [developed also in different versions by Halle and Marantz (1993), Hale and Keyser (2002), Borer (2005a,b, 2013), Ramchand (2008, 2018), and Londahl (2014)], for the sake of exposition here, Nanosyntax (Caha, 2009; Starke, 2009).

Our choice for selecting exoskeletal variants of Minimalist grammars, Nanosyntax in particular, boils down to the interesting contrast they provide when stacked up against CxG. With regards to their architectural similarities, both adopt the position that the internal composition of larger elements are determined by the structural conditions (or “frames”) they appear in. In both cases, the claim is that the lexicon contains information about structure, in some sense, either as templates or as configurations. In contrast, these two are diametrically opposed to one another in two fundamental ways: first, exoskeletal grammars – and Nanosyntax in particular – adopt the stance that all complex mental representations must (always) be built, and that the computational system should proceed “with as small as possible a repository of idiosyncratic information appended to it” (Borer, 2005a:15). Second, whereas most versions of CxG are declarative and model-theoretic, exoskeletal grammars adhere to a derivational, proof-theoretical system. As we demonstrate throughout the remainder of this essay, the desiderata employed by these two frameworks highlight and contribute to a host of other related and important issues closely connected to debates circling around the nature of the human mind.

One well-known, traditional way of making a distinction between these two frameworks depends on whether any sort of domain-specific properties of language exist and are drawn upon to aid language development and language processing (often referred to as Universal Grammar; UG), or whether domain-general mechanisms are solely responsible for the creating of linguistic constructions [often referred to as a usage-based approach; see e.g., Bybee (2010); see Adger (2019) for an excellent discussion and overview of this debate]. Although our treatment of the definition of constructions from these two perspectives below certainly touch upon this critical divide, diving too deep into this immediate debate would detract from our discussion of the structural properties of constructions, and so we acknowledge the secondary role this ongoing debate plays here. That point notwithstanding; however, it behooves us to point out that although domain-general cognitive properties undeniably do effect outcomes in language acquisition and development across the lifespan, a direct association between linguistic outcomes and psychological embodiment is highly controversial with regards to phonetics and phonology^1^ (Berent, 2013; Berent et al., 2020), syntax (Tettamanti and Moro, 2012), and semantics (Meteyard et al., 2012). The cumulative sum of this research is echoed in Aroud (2020) plea for more attention and focus on the notion of mental representations, which also supports Jackendoff (2017) reminder of the central theoretical importance of theories of mental (linguistic) representations. Thus, irrespective of one’s position on the existence and role of domain-specificity in the generation of linguistic structure, the very nature of mental representations is of vital importance.

The position set forth in this paper is the following: even within frameworks whose main tenet is that the primitive units of analysis lack any degree of internal composition, there are non-trivial notions of ‘composed unit’ that could be characterized as constructions, as they have some of the crucial properties of these types of elements. This article, then, argues that constructions and construction-like units are identified at multiple scales from the very beginning. Thus, even if it turns out that the smaller units are not unanalyzable primitives, some kind of bottom-up structure building is required from the start. Ultimately, therefore, understanding the nature and properties of the building blocks of language is a common enterprise unites us across cognitive disciplines and frameworks. The structure of this essay is as following: In see section “Constructions: Decomposition and Composition” we establish the fundamental established criteria of the notion of construction, primarily from the perspective of a domain-general tradition (as opposed to a domain-specific one). In see sections “Constructions in Minimalist Grammar: Semantic Interpretation and The Role of Constructions in the (Morpho)Phonological Interpretation of Objects,” we discuss the role of Constructions according to Nanosyntax (and Minimalist parlance more generally). Adopting an exoskeletal approach to grammar which espouses with the notion of a pre-syntactic lexicon, we maintain that the notion of construction proper is best understood as a second order units that appear at the interface of syntax and its interface with Phonological Form (PF) – the modular domain of grammar responsible for morphophonology. Section “Conclusion” concludes this essay.

## Constructions: Decomposition and Composition

Constructions are defined as form-meaning mappings, potentially containing combinations of independent units, whose meanings and grammatical properties are not predictable from any identifiable internal composition (Goldberg, 2006). One of the more salient consequences of this idea is the possibility of representing (storing and processing) unanalyzed chunks at a fairly large scale, e.g., that of an entire sentence. However, this doesn’t imply that constructions are always large, nor that they cannot be decomposed into smaller units. Rather, it emphasizes that the meaning of a complex linguistic unit is, or at least can be, more than the sum of whatever smaller units and structure can be identified therein. This view also does not deny that meaning can be compositional. The meaning of an utterance may involve combining the meaning of a construction with that of lexical items or phrases occupying variable slots, or with other constructions, for example. Thus, meaning maps onto linguistic form at multiple scales simultaneously, and meaning is partly compositional, and partly not.

As we will argue below, this picture shares much with more traditionally generative approaches, which, despite their strong tendency to view meaning as derived from structural organization, must nonetheless acknowledge the existence of units with discernible internal structure whose meanings are not reducible to the sum of their parts (in ways that go beyond mere lexical idiosyncrasies, e.g., idioms). Where these approaches diverge is in the theoretical handling of units at different scales. CxG is an example of an emergentist approach to grammar, where units and their combination are defined on the basis of salience to the user given their prior experience, learning and processing mechanisms, and current conditions.

This can be understood at both the developmental and the historical scale (e.g., Tomasello, 2003; Bybee, 2010; Christiansen and Chater, 2016). Developmentally, children identify, create, and store chunks that are discernable in their experience, which may be reused whole, and gradually learn to decompose and compose them, based on the recurrence of similar material in different contexts. Parallels among larger chunks allow children to discern abstract structure that permits chunks to be decomposed into smaller units and recombined productively, and the frequent co-occurrence of smaller chunks leads to the identification of larger functional units, including not only collocations but also syntactic phrases (Hopper, 1998; Langacker, 2000; Bybee, 2001). Note that this does not imply that children begin by memorizing larger (e.g., utterance- or intonation unit-sized) chunks, nor that the emergence of structure always moves from larger-scale units to smaller component units. Whatever units the learner can initially identify from experience, be they syllables, morphemes, words, or larger constructions, are subject to processes that both permit them to be analyzed into smaller units or unitized into larger ones (for a computational implementation of this bidirectional process see McCauley et al., 2015). Historically, language change occurs in a similar way as shifts in the ever-present variability of usage patterns either obscure old patterns or allow new ones to be identified (Bybee, 2010), and as these changes in usage are filtered through the learning and processing mechanisms of succeeding generations (Fedzechkina et al., 2012; Culbertson and Newport, 2015). Change may also occur as individual users age, because the accumulation of experience can lead to changes in the demands that language places on cognitive processes (Ramscar et al., 2013).

Perhaps the most important consequence of this emergentist view of structure is that the units into which constructions may be decomposed are not defined *a priori*, at least not from the perspective of the individual learner. Instead, they are identified gradually and piecemeal as the learner’s experience permits, eventually approximating the grammatical system of other individuals with comparable language experience. In this sense, individual learners are not credited with possessing *a priori* units or structures, but we may nonetheless speak of *potential* units and structures, inherent in the usage patterns of the community and learnable through the operation of general cognitive mechanisms on patterns available to the learner through experience. This does not, in principle, exclude the existence of some innate knowledge of the kinds of structures children are looking for, although the usual approach is to avoid assuming it, and as we stated above, we consider this question to be a secondary issue. This approach to an “open UG” has also gained traction in generative approaches to acquisition and language change (Lightfoot, 2020).

The main question from a CxG-perspective is not, therefore, whether constructions possess internal structure. They do, in the sense that members of the community produce language instantiating patterns describable in terms of smaller-scale units and patterns (rules, constraints, or whatever) for combining them, and in the sense that the presence of non-compositionality does not preclude a role for subunits in the real-time processing of language. Rather, CxG, and emergentist approaches in general, focus on when those patterns play a role for the learner or speech community, and when they do not.

This can be thought of as akin to Yang’s Tolerance Principle, where the balance of regular and irregular structures in the learner’s repertoire determines whether or not the regular rule is operative, or whether items containing potentially regular structures are represented and processed as unanalyzed wholes (Yang, 2016). Thus, for example, Yang proposes that children may represent regular English past tense verbs either as a *stem* + *ed* combination, or as an unanalyzed word, depending on how many regular and irregular past tense wordforms the child has learned. This offers an explanation for U-shaped development: at first, children produce few over-regularizations, because they are producing both regular and irregular verbs as memorized wholes. Then as the rule/pattern strengthens, they produce overregularization errors (e.g., *eated*), and finally they sort out the regulars and irregulars, processing the irregulars as unanalyzed wholes, and the regulars as *stem* + *ed* combinations (Tomasello, 2003). The Tolerance Principle, based on psycholinguistic understanding of lexical processing, helps explain when the rule becomes available. A similar phenomenon can be identified in the ways that type and token frequencies shape language change (Bybee, 2010). Forms and patterns that are frequent enough to be memorized tend to be stable, but infrequent forms may be adapted to fit robust patterns (e.g., paradigm leveling), and patterns with low type frequency may lose their productivity.

A second major consequence of emergentism is to further develop the character of what it means to learn or know a grammar. Language acquisition is not conceived as a search for the right set of units, and the rules or constraints that govern how they are used in a particular language, either in the sense of discovering them from experience or of narrowing down a set of innate structures. Rather, to learn a language/grammar is to learn to *process* language (Chater et al., 2015; Christiansen and Chater, 2016). It may be that, from the standpoint of a speech community, where individuals’ experience of language can be expected to be relatively consistent, we could (in the limit) identify an exhaustive set of potential units and patterns that are *available* to be learned, but from the point of view of the individual user in the moment of producing or perceiving language, there could be many ways in which a piece of language (say, an utterance) may be represented for processing. Put differently, what’s important is not the maximally articulated structure that *could* in principle be used to represent the utterance. The important question is what representations and structure the individual user has at her disposal, and which ones, from that repertoire, she *does* use on a particular occasion of language use, from a single undecomposed unit, to a detailed, hierarchically organized set of smaller units. The way this shakes out on any given occasion is determined in large part by the cumulative prior experience of the learner, and the specific abstractions that this experience permits to emerge given the operative learning mechanisms, and it is also subject to real-time conditions, including properties of the preceding discourse, prosody, familiarity with the topic or interlocutor, or cognitive load.

As a useful illustration of this state of affairs, consider the last several decades of psycholinguistic research on morphological processing. To sketch this history only very briefly, an early debate centered on the decomposition of complex words into stems and affixes. Evidence that non-words like *juvenate* and *dejuvenate*, which contain stems that occur in prefixed words like *rejuvenate*, are harder to classify than non-words like *pertoire* and *depertoire*, which do not (the *re-* in *repertoire* is not a prefix) was taken to indicate obligatory decomposition in the processing of complex words (Taft and Forster, 1975). Later work interpreted effects of the whole-form frequency of complex words as evidence that at least some complex words are stored as unanalyzed wholes, but not necessarily precluding decomposition as well (Schreuder and Baayen, 1995; Baayen and Schreuder, 2000), with evidence that a whole-form representation might be formed on the very first encounter with a new complex word (de Vaan et al., 2007). Still more recently, the observation of whole-form frequency effects even in very low frequency complex words (Baayen et al., 2007), and interactions among whole-form and constituent frequencies (Moscoso et al., 2004; Kuperman et al., 2010) have been interpreted as indicating the simultaneous and integrated processing of both holistic and compositional structure.

It would be tempting now to propose a rapprochement, by pointing out that CxG no more denies the presence of smaller units than exoskeletal approaches do the existence of larger units. That compositionality is required even in a Construction-theoretic paradigm has never really been in question, and we will argue shortly that something like constructions are not only possible, but required in a more traditionally generative view as well. With this agreement, we can perhaps work out what all of this means for why human language has developed ontologically and phylogenetically the way that it has.

But this seems a bit glib, and obscures the point where things actually get interesting. Namely, how are we to explain why humans consistently come up with extremely similar ways of representing language? This is true not only of the members of specific speech communities, where statistical distributions over a relatively common corpus of linguistic experience can go impressively far in identifying the units that members are sensitive to, or will become sensitive to given sufficient experience. It is also true of the major commonalities observed across languages around the whole world, and those from the past that have left a written record. That is, humans across time and space appear to have much in common, including what appear to be common categories of representations that are used in similar ways, with an apparently limited range of variability. Explaining this can be thought of as the overarching goal of any scientific approach to human language, and we return to this below as the really exciting way to explore a synthesis of emergentist (represented by CxG) and generativist (represented by exoskeletal) approaches. At this point, however, we turn to the question of constructions in Minimalist Grammar.

## Constructions in Minimalist Grammar: Semantic Interpretation

The title of this section is intentionally provocative. Our goal here is to argue that, implicitly, even Minimalist accounts (Chomsky, 1995) have an implicit notion of construction that is assumed in most linguistic analyses, at least in a weaker sense. This happens in two situations, (1) one affecting the semantic properties and (2) another affecting Spell-Out: whenever the semantic properties of a syntactic object are not interpreted as soon as possible, that is, as soon as structure-building operations such as Merge create a new structural layer, or when the exponent corresponds to a complex constituent rather than to one single terminal. In this section we will concentrate on the first situation, and we will discuss the second in the following one.

Minimalism proposes that the computational system builds up complex structures by adding one unit at a time, so in this sense there is no notion of construction. However, construction-like objects emerge when one considers the interpretation of those structures in semantics. If the set formed when X and Y are merged is fully interpreted as soon as Merge happens, no chunk of structure, i.e., “construction,” is needed for interpretation; however, if the combination of X and Y is not semantically interpretable, and must be postponed until a second layer is built, then we must conclude that interpretation applied to a chunk without applying to each one of its internal constituents. This is clearly reminiscent of the CxG tenet that the notion of construction is the domain where meaning is defined, even if, to be fair, Minimalism treats the satisfaction of meaning compositionally, while CxG is not necessarily committed to a notion of compositionality. For this reason, this implicit notion of construction is weak in Minimalism; however, constructions are properly understood as derived objects rather than theoretical primitives. That is to say, Minimalism does not allow constructions as primitives of structure building, but rather they emerge, similar to what is claimed in CxG, when structure is built.

To illustrate what we mean, consider (1) and (2), which are different types of structures that require assigning interpretation to chunks. They both violate in different ways the principle of “assigning interpretation to each unit locally.” In (1), the violation is weaker, because it could be avoided if we assumed that “local” means “within its own XP projection.” If within XP the meaning of X is satisfied, one can assume that there is a particular complete semantic object {S} that at LF would stand for XP [see Chomsky, 2013, where it is argued that labeling is required only at the interfaces, i.e., at the juncture when structural objects are “interpreted” for semantic compositionality (LF) and for the realization of morphophonology (PF)]. That is: one single element, XP, corresponds to a particular semantic object, for instance a particular predicate with its arguments satisfied. Assume, for the sake of the argument, that X equals V and (1) represents a (rudimentary) VP. X is a predicate that would select two arguments – whose place-holders are *a* and *b*–. (1a) represents this in semantic notation, using lambda abstraction that expresses the two open variables; (1b) represents the same in syntax, with the two placeholders corresponding to two structural positions.

(1)
  a. λbλa[X(b,a)]
  b.    [_X__P_   [a]   X   [b]]

Importantly, even in (1) there would be an intermediate structure-building operation where the head X is still not semantically satisfied, so in the strict sense one would have to consider that there is a local step that does not get interpretation.

The derivations in (2) represents a stronger violation of the principle that interpretation should be as local as possible. Imagine that the satisfaction of the two variables related to X takes place within a bigger chunk of structure, one that goes beyond XP, as in (2b) or even (2c).

(2)
  a. λbλa[X(b,a)]
  b.    [_Y__P_    [a]   Y   [_X__P_   X   [b]]
  c.    [_Y__P_    [a]   Y   [_*Z*__P_       [b]   Z   [X]]]

Now, from the perspective of semantic interpretation (LF), the well-formed semantic object will not be X, but rather YP, which contains XP and possibly ZP. The semantic interpretation of X – in our example, a predicate – would correspond to YP, a bigger structural constituent that contains X and additional heads and members.

Semantic interpretation is compositional and local if, and only if, any combination of two items in syntax is interpreted semantically at LF. Any node of information organized hierarchically in the tree, then, would have to get an interpretation assigned. Postponing interpretation to a further syntactic structure-building operation automatically implies that a chunk of structure containing two or more layers is, in that context, the smallest object that can be assigned an interpretation. Any such case would be a “construction,” again in the weak sense.

The ultimate motivation for this idea that requires every operation which builds a new structural layer should be interpreted is the so-called Frege’s conjecture. The conjecture is, in essence, that natural language builds complex meaning always through the same procedure: function application. Syntactic combinations such as (3), where a head takes a complement and labels the resulting set, must be invariably translated into semantics as the head being a function that takes the complement as its argument.

(3) [XP   X   [Y(P)]]

If function application is the only available operation; the following iteration of the structure-building operation should mean that now XP is the argument of a function introduced by Z. Thus, the properties of X must have already been satisfied within XP, as there is no possibility that X still is a function that takes Z as an argument, for instance.

(4) [    Z   [XP   X   [Y(P)]]]

Minimalist grammars do not shy aware from this problem, and in fact its existence guides some explicit proposals about how syntactic structure should be mapped into semantics, precisely to guarantee as much as possible that the interpretation of a head X is satisfied within its own projection. Pietroski (2018) monograph is an attempt to set the basis for a purely conjunctional approach to complex meaning which satisfies this no-chunk requisite. Londahl (2014) applies this type of analysis to the building of verbal eventualities in an explicit and convincing way, dividing what we take, at least on the surface, to be one single predicate into an n-number of heads each one devoted only to one particular semantic layer.

To be clear, not even (1) complies to Frege’s conjecture if every step of the syntactic-building operations must be interpreted: in (1), the intermediate step where X has combined with b and a has not been merged yet would not correspond to a semantically well-formed object yet, so interpretation would have to skip this step and be postponed until the whole XP is closed – therefore, the interpretation would apply to a chunk of sorts, in this strict interpretation of compositionality.

Complying to Frege’s Conjecture has been viewed as a desideratum of syntactic analyses, and in particular within Minimalism. Proposals such as Pietroski (2018) and Londahl (2014), therefore, make sense as explicit attempts to avoid the chunk-problem that we just mentioned, even in the form that (1) presents. This, of course, amounts to admitting that the problem is real, and that if minimalist syntactic analyses allow for correlations between syntax and semantics of the type of (2) they must make room for a weak notion of construction that is undesirable in the strictest sense of establishing a heuristic of “locality.” Analyses along the lines of (4) are, thus, to be preferred all things being equal, and we believe that it is fair to say that any minimalist approach would attempt to come at least as close as possible to (4).

However, and as usual, reality is stubborn and it is unclear how, or even whether, every single structure in syntax can be codified in structures that satisfies Frege’s Conjecture. One possible objection that comes to mind in this respect is idioms, which require at least parts of their meaning to be built syntactically [see McGinnis (2002) for similar arguments]. However, in the case of idioms one could argue that what makes them special with respect to meaning should be located at the lexical level, the domain of conceptual and world knowledge meaning, in a way that their idiosyncrasies would not directly interfere with how syntax is mapped to LF. There are, however, many other syntactic structures that are problematic from this respect.

One empirical domain where the mapping between syntax and semantics has been particularly problematic form the perspective of Frege’s Conjecture is the analysis of comparative structures. Take an example like (5).

(5) Covid-19 is more dangerous than the regular flu.

The very abundant literature on the semantics of these constructions (see Klein, 1991; Schwarzschild, 2008; Beck, 2011 for distinct overviews) agrees on two facts, beyond many controversies. The first fact is that the adjective, here *dangerous*, must contain some type of open variable corresponding to degree – the extent to which an entity exhibits the property denoted by it. The second is that the adverb *more* is somehow assigning a value to that open degree variable by identifying it with those values higher than the degree of dangerous exhibited by the regular flu. Semantically, this corresponds to (6) for the semantics of the degree adverb, and to (7) for the semantics of the gradable predicate.

(6) λyλPλx∃d,d′. max[λd.P(d,x)] > max[λd′.P(d′,y)]
  (there are two degrees d and d′ such as that for a property P, the maximal degree d of P that x has is higher than the maximal degree d′ of P that y has)

(7) λdλx[x is d-property]
  (the subject x has the property to a certain degree)

The question is how this semantic denotation is represented in a syntactic structure. Note that according to (6), the degree adverb takes three arguments: a property (that is, a predicate) and two entities that display that property to different degrees. Larson (2014): 471), being completely aware of the compositionality problem, proposes that the degree adverb is in fact the head of the structure, taking as complement the second member of the comparison and as its specifier the property. The semantic properties of the comparative are almost completely satisfied within one single XP, except that Larson (2014) proposes that degree should be divided in two related heads, using the highest one to introduce the subject of predication of the adjective (see also Bowers, 1993; Baker, 2003 for the problem of where the subjects of adjectives are introduced). In any instance, if the *v*P-shell structure is actually viewed as the projection of two essentially identical categorial heads, this type of structure would satisfy Frege’s Conjecture: the head is a function that takes other items as its arguments.

(8) [_Deg__P_ [pro] Deg [_Deg__P_      [_*AP*_ dangerous] Deg   < more > [_PP_ than the flu]]]
The lower Deg head would head-move (Travis, 1984) to the higher one, producing (9).
(9) [_Deg__P_ [pro] Deg < more > [_Deg__P_ [_*AP*_ dangerous] Deg <more> [_PP_ than the flu]]]

At LF, the head DegP would be translated as a saturated predicate where the subject *pro* would exhibit a property to a particular degree. The problem of this structure, from the syntactic point of view, is that it is not compatible with the standard assumptions about head movement. Consider the cases in which degree is not expressed through the morphologically free adverb *more*, but through the semantically identical suffix -*er*.

(10) Covid-19 is deadli-er than the regular flu.

In Larson’s structure, the head of the AP must rise from a specifier position to the higher head, in order to get combined with the suffix. This movement operation is illegitimate given standard assumptions. For this reason, an alternative account that is more popular among syntacticians less concerned with semantic compositionality is (11), where degree is a functional head that projects above the lexical layer AP (see for instance Corver, 1997).

(11) [_Deg__P_   Deg < more/-er >      [_A__P_   dangerous/deadly]]

Independently of the position of the subject of predication, this structure now faces a problem in terms of semantic compositionality. There are two options with respect to where the comparative coda *than the regular flu* is introduced, and both force the conclusion that the semantics is satisfied within a chunk of structure that exceeds the domain of the head that defines the function. The first option is to introduce the PP coda as the specifier of DegP (12).

(12) [_Deg__P_    [_PP_ than the regular flu] Deg < more/-er > [_A__P_ dangerous/deadly]]

The fundamental problem with this structure is that it implies an intermediate step where the degree that *more* identifies is defined as higher without specifying what reference value is used to define what counts as higher. Specifically, that would be the Deg projection before merging the specifier, whose denotation would be (13).

(13) λyλPλx∃d,d’. max[λd.P(d,x)] > ?
  [Deg   [AP]]

Interpretation would then have to be postponed until the following layer of structure is built, just as we said was the case with (1), with the result that there would be a structure-building operation that does not get interpreted: interpretation would have to be postponed until the second structure-building operation involving Deg. The second option is even more clearly against Frege’s desideratum: it would imply merging the PP coda within the AP.

(14) [_A__P_    dangerous [_PP_ than the regular flu]]

However, this goes against the interpretation of a gradable adjective as presented in (7). Specifically, licensing the comparative coda would have to wait until the specific degree element is introduced in the following layer. Either way, the interpretation would not be satisfied until additional layers of structure are built, and cannot happen at each step in the derivation, as Frege’s conjecture would require.

The conclusion is that, in the current state of knowledge, the syntactic structures required to capture some of our standard assumptions force us to accept a weak notion of construction where we have to admit that there are intermediate steps of the structures that cannot receive an interpretation, and complex chunks receive the interpretation instead.

## The Role of Constructions in the (Morpho)Phonological Interpretation of Objects

In the same way that it is not always the case that the semantic structure can be read at each single step of a hierarchical syntactic tree created by iterative applications of Merge, standard Generative Syntax also accepts that in some circumstances the PF materialization of syntactic elements also has to apply necessarily to chunks of structure. This is, after all, what underlies the empirical phenomenon known as cumulative exponence (Spencer, 1991; Stump, 1998, 2001). Cumulative exponence is the situation where one single morph, sometimes called a “portmanteau morph” (Hockett, 1958), materializes information that has been independently diagnosed to be contained within two or more syntactic unit, standardly understood as heads according to Minimalist parlance. In this section we approach the syntax-morphophonology interface from the perspective of Nanosyntax (to be discussed below).

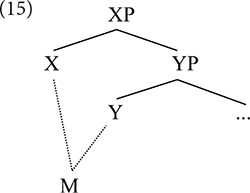

Irrespective of how this phenomenon is analyzed, cumulative exponence implies that at some level the morphophonological information that is associated with the morphosyntactic features must be taken into account not at each separate layer of structure, but rather as representing a combination of at least a minimum of two layers. Portmanteau morphs are uncontroversially illustrated by the case of exponents that materialize the set formed by some prepositions with certain determiners, as in French (Haugen and Siddiqi, 2016; Svenonius, 2016).

(16)
  a. ^∗^à le vin
    to the.sg wine
  b. au vin
    to.the.sg wine
    “to the wine”

(17)
  a. ^∗^à les voyageurs
    to the.pl travelers
  b. aux voyageurs
    to.the.pl travelers
    “to the travelers”

It is uncontroversial that the preposition and the determiner must constitute distinct structural layers in syntax (18) – perhaps intermediated by additional heads beyond those expressed in the tree.

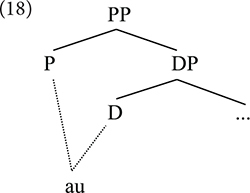

Therefore, the exponents *au* and *aux* illustrate a situation where PF must consider a structural chunk bigger than one single unit to introduce the right exponent. Crucially, only some prepositions will require this cumulative exponence; example (19a) and only some combinations of gender and number in the determiner would trigger it (19b vs. 16a), which shows that PF must be sensitive both to the individual exponents that could have been used in each separate head and the syntactic information contained in them.

(19)
  a. avec les voyageurs
    with the travelers
    “with the travelers”
  b. à la mode
    to the.f fashion

Current theories have a variety of technical procedures, dependent on their broader theoretical tenets, to address these cases. Word-and-Paradigm morphological theories (Robins, 1959; Matthews, 1991; Stump, 2001; Spencer, 2013) propose that morphemes are not proper units of analysis and the materialization of morphosyntactic features takes place at the word level, intermediated by rules that associate specific word forms with specific sets of features that, syntactically, can be dispersed among several heads. Distributed Morphology (Halle and Marantz, 1993; Siddiqi, 2018) proposes several procedures to account for portmanteau morphemes, including PF-rules that map distinct layers of structure in one single position of exponence and readjustment rules that reorganize the information contained in distinct syntactic positions in the presence of specific exponents. Ramchand (2008, 2018) and Svenonius (2016) propose a spanning procedure that spells out a sequence of distinct heads into one single exponent, with a non-trivial notion of “word” also defined in the second case through diacritics that impose that all heads contained within a chunk of structure are spelled out as part of the same morphological unit. Nanosyntax (Caha, 2009; Starke, 2009) adopts a phrasal spell-out procedure whereby exponents do not need to be introduced in terminal nodes, but can actually substitute XP constituents, including specifiers and complements; see also Fábregas and Putnam (2020), who uses phrasal Spell-Out but allow exponency to be defined at a level distinct from both syntax and PF. Leaving the technical distinctions aside, the fact is that in all these theories the materialization of a portmanteau morpheme necessarily must take into account the information provided by a complex chunk of structure. The procedure that maps the information contained in a single syntactic node to an exponent cannot function simply by looking at the information contained in that node: it needs to consider (depending on the theory) the whole set of features spelled out in the paradigm, the syntactic heads above or below it or the XP configuration where a single head is located. The result of this is that in the generative tradition – but not in CxG – the relevant notion of “construction” is a second order unit used to associate additional information to the abstract syntactic structure, which is in turn derived principally from a limited set of universal operations that insure the well-formedness of linguistic structure.

As in the case of the mapping between semantics and syntax, this problem has been noted, although it has not been considered as serious as the previous one – perhaps because current generative theories interpret PF as a highly idiosyncratic level of representation that might not be subject to the minimalist principles identified in syntax–. In Nanosyntax, one technical device that addresses this issue in part is the notion of “pointer” (see Starke, 2009; Caha and Pantcheva, 2012; Caha, 2018). A pointer is a device that, within one lexical entry, refers to another lexical entry. Consider one example. Assume a simple syntactic structure like (20).

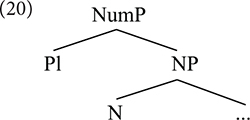

Imagine that the first head, N, is spelled out somehow as *mouse*. Once that layer is spelled out, or lexicalized, the Spell-Out operations applies to the second layer, but of course the spell out of [Pl] in this context would not be -*s*. Because of the lexical item introduced as N, its lexicalization would be irregular, as *mice* (21a). In a nanosyntactic system with pointers, the lexical entry of (21a) would look like (21b), stating specifically that that exponent is materializing the lexical item *mouse* in the context of plural number.

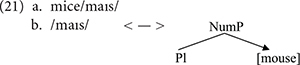

The entry in (21b) stores a “phonological idiom” of sorts, which blocks the materialization ^∗^*mouse-s*, where each layer is spelled out independently. Instead of simply adding -*s* as the spell out of [pl], (21b) replaces the lexical item *mouse* with *mice* in the context where it appears under plural number. The hierarchical organization of linguistic information turns out to be a non-trivial architectural design feature, as it provides a systematic way to make predictions regarding how syntactic structures shape the lexicalization of these structures at PF (Embick and Marantz, 2008). This design feature; namely, the requirement that syntactic information is hierarchically organized is not emphasized in most variants of CxG. For example, Jackendoff and Audring (2020), in spite of arguing for a tripartite structure of “lexical items” consisting of semantic interpretation, syntactic structure, and morphophonology (similar to what is maintained in Nanosyntax), call for a parallel, rather than a hierarchical architecture of syntactic representation. On the other hand, the chunk-and-pass nature of language processing described by Christiansen and Chater (2016) can be thought of as a hierarchically organized representation of utterances in the form of a processing trace. An reviewer raises the question as to whether these particular design features in the exoskeletal approach can be incorporated into experimental research, leading to predictions that would illustrate how this architecture is potentially superior to CxG-approaches. Such work, in fact, does exist, showing the empirical advantage of redefining the notion of the traditional “morpheme,” understood as a stored unit of sound and meaning (much like a “construction”), to include abstract hierarchical structure facilitates processing gains (Marantz, 2013; Gwilliams, 2019).

The pointer in (21b) alleviates the need to introduce chunks as units of Spell-Out, but it does not solve the problem entirely. A pointer makes it possible to make direct reference to a lexical entry within a lexical entry, allowing the Spell-Out procedure to apply at each layer of structural complexity: instead of having to wait until [pl] is spelled out to spell out the whole chunk, the NP-layer can first be spelled out, and then the spell out of [pl] overwrites the previous lexical item because the lexical entry has a pointer referring to it. Thus, in this system spell out can happen at each single layer, without having to consider complex chunks as units. However, reference to a chunk is still needed in order to select the portmanteau exponent in the second instance of Spell-Out. The chunk is needed to introduce, and realize, exponents, even if Spell-Out applies sequentially at each layer: we have not removed the chunk, just changed the level of representation where it is relevant. In toto, we believe that, just in the same way that chunks are necessary in establishing the connection between structure (i.e., syntax) and meaning (i.e., semantics), they are also necessary at PF to spell out structures. Exoskeletal variants of Minimalism, such as Nanosyntax, possess the necessary tools to derive both idioms and larger structures (i.e., constructions) with arguably only minor necessary adjustments to structure-building operations such as Merge. An implementation of this principle can be found in Fasanella and Fortuny’s (2016) *Chunking Procedure*:

(22) Chunking Procedure:
  Given a head *H*, the learner determines:
  a. whether *H* is phonological dependent of other heads ([ + bound]) or not ([-bound]),
  b. whether *H* conveys only one morpheme ([-synthetic]) or more than one morpheme ([ + synthetic])

Assuming an architecture of syntax in which each functional head consists of one and only one feature (as is the case in Nanosyntax), the learner will be able to detect how a given language encodes information as minimal units (such as morphemes) or whether or not additional structure may be required. Returning to our previous discussion of cumulative exponency, learners must acquire the knowledge whether for a given category the grammar they are acquiring prefers the setting of [ + synthetic] for a particular form-meaning-sound pairing. This fits the basic criteria of construction introduced in see section “Constructions: Decomposition and Composition” of this paper and represents that essential empirical cue that will leads to the successful acquisition of this attribute of the grammar. Although experience will ultimately determine the parametric settings that differentiate one language from another, an exoskeletal architecture with only two proposed binary axioms can effectively govern and shape this acquisition process. These first order operations and the way they determine how syntactic structures (i.e., representations, or “constructions”), are for principle concern of generative linguists.

Ultimately, both CxG and Nanosyntax acknowledge the existence and important role that “constructions” play in language acquisition and development. In this respect, one could boldly state that certain aspects of these respective research programs are mutually supportive of one another, and that there are even avenues of research in which one could envisage some form of collaboration between scholars from both traditions.

## Conclusion

In this abbreviated positional essay we have attempted to highlight substantial areas of convergence between generative and emergentist traditions while at the same time acknowledging points where they (critically) diverge from one another. Taking stock of the discussion above, we ascertain that both approaches adopt the following position on constructions/chunks:

(22) Points of agreement
  (a) In both domain-specific and domain-general approaches, constructions are interpreted as specific domains for interpretation, both with respect to semantic and morphophonological information, that in principle can exceed a single terminal, and
  (b) Constructions must be “fixed” (at least to a certain extent), in the sense that they must be related to a particular representation which contains some invariable elements. These invariable elements may consist of specified morphophonological representations, the requirement of a particular semantic interpretation, or even the assignment of specified grammatical classes.

In turn, we highlight several particular domains that are still disputed by researchers who ascribe to one of these two camps:

(23) Points of divergence
  (a) The degree of idiosyncrasy that constructions may contain. From the perspective of Spell-Out in a derivational model such as in Nanosyntax (and Minimalism more generally), domain-specific frameworks accept that the relevant chunk is idiosyncratic in the sense that the information that these units add is not predictable from the properties of the terminals that they contain. In this sense a spelled-out chunk is not different from a spelled-out single terminal, because in both cases the assumption is that the morphophonological entries are not motivated. In contrast, with respect to semantic interpretability, Nanosyntax still expects compositionality to apply – even if the full interpretation of some elements of structure (i.e., heads) are not satisfied locally, within the chunk it should still be traceable which part of meaning each one of the heads contributes to the whole. CxG does not commit to compositionality in this sense and allows for a system where the semantic interpretation is entirely due to the construction without the possibility of determining which internal component carries which portion of meaning.
  (b) Their extensions across levels of grammar. Minimalism and other generative approaches do not accept construction-like units as primitives, but rather as devices that in some cases are necessary to account for the semantic and phonological properties of those structures. In contrast, CxG takes constructions as the minimal building blocks, i.e., the construction itself gives a template that defines some structural properties. In this sense, one could argue that in Minimalism and other generative approaches, construction-like units are second-order objects used to associate the syntactic structure with the information of other levels – semantics and phonology – but never centrally involved in building the structure itself. While one can say that construction-like units are second-level objects in Minimalism, this is not the case in other traditions, that put usage at the center and thus use constructions as building blocks for production and comprehension.
  (c) Their stored or derived nature. Late-insertion approaches where one exponent corresponds to more than one unit tends to advocate for a view where the chunk corresponding to an exponent or a particular semantic interpretation is not stored, but actively built – derived – anew by structure-building processes such as Merge (see Embick, 2015 for a detailed explanation of this position). Even when the exponent corresponds to a chunk and therefore must be listed, the chunk that it replaces through PF-insertion (i.e., the realization of morphophonological material associated with a particular chunk/structure) is not stored: the computational system builds it up from specific units and the relevant exponent(s) that correspond to the resulting structure are introduced when the structure interfaces with Phonological Form (PF). In CxG, the construction is pre-assembled in a sense, because constructions are stored and they themselves correspond to the relevant level of structure that is used to produce and interpret linguistic sequences. Nevertheless, though they are stored, constructions may also be decomposed into smaller units, depending on their relationships with other constructions in the user’s experience, and in this sense they can be thought of as simultaneously first-order and second-order objects.
  (d) The existence of a set of primitive units, from which all others are derived. It is not so much that generative grammar argues for, and CxG against, this idea. Under CxG, the units by which language is represented are those that are accessible to the user at the time of processing, based on her prior experience and current conditions. At most, it might be possible to identify a “most finely articulated” parse of a given utterance, that anyone with sufficient experience might achieve. The structure of this parse could be understood as the product of the structure of the world (e.g., cause-and-effect, the flow of time) and the properties of human cognition, even including domain-specific adaptations in the human mind (which we identified as a secondary issue at the outset of this essay), given sufficient reason to posit their presence. However, since units of any scale are held to be emergent, there is no reason to expect any universal, species-wide inventory. Whatever deep commonalities there appear to be across cultures and history, they may be too general to explain the richness of linguistic structure (e.g., not much mileage comes from the observation that we all appear to have a Noun category). Of greater consequence, even if we could identify a set of units that would yield a most basic parse of any utterance, an emergentist view (like CxG) offers no reason to think of this as any kind of an endpoint toward which all users of that language are headed. Language development is not held to be linear, and the optimal use of language sometimes requires that finer-grained structure be ignored (Plag and Baayen, 2009).

The plea for more attention to mental representations (Arntz, 2020) requires us to revisit the central role that the structure of language plays in attaining a better understanding of its ontogenetic and phylogenetic development in our species (Stroik and Putnam, 2013). The concept of construction is certainly a loaded term, with different camps of linguists and language scientists adopting diverging definitions of these units and the role that such items play in these respective research programs. There are indeed attempts to unify aspects of these programs, or at the very least, address how the architecture of some versions of formal grammar may be mutually compatible to both camps, at least to a certain degree (see especially Culicover and Jackendoff, 2005 and Trotzke and Zwart, 2014). In a sense, we are arguing for a “yes-both” approach to the question of mental representation of language, which will necessitate further work on the conceptual basis for theory, leading to testable predictions.

To this end we propose a series of questions that may lead to greater synergy among Exoskeletal and CxG, Emergentist and Minimalist, and domain-specific and domain-general approaches to language: (1) If CxG allows for the emergence of decomposed representations of constructions as language development progresses, what are the consequences for the notion that the construction is a primitive unit? Can constructions come to behave as second-order units with regard to the mental representations in play for specific language users at specific times? (2) How shall we characterize the (maximal?) potential structure available to members of a speech community, given enough experience?, and (3) How universal is that potential structure across languages, and what is the source of this (apparent?) universality? In our view, exploring these questions will bring together researchers from the two traditions in the common enterprise of understanding the human capacity of language, and, whichever the answers ultimately are, in a more comprehensive view of human cognition.

## Data Availability Statement

The original contributions presented in the study are included in the article/supplementary material, further inquiries can be directed to the corresponding author.

## Author Contributions

AF took the lead on sections “Constructions in Minimalist Grammar: Semantic Interpretation” and “The Role of Constructions in the (morpho)Phonological Interpretation of Objects.” MC on section “Constructions: Decomposition and Composition.” MP sections “Introduction” and “Conclusion.” All authors contributed to the writing and revision of this manuscript.

## Conflict of Interest

The authors declare that the research was conducted in the absence of any commercial or financial relationships that could be construed as a potential conflict of interest.

## Footnotes

1 The zero-sum of the Berent et al. (2020) work shows that phonetics could easily be associated with embodiment but this is certainly not the case for phonology.

## References

[B1] Adger D. (2019). *Language unlimited: The science behind our most creative power.* Oxford: Oxford University Press.

[B2] Arntz A. (2020). A plea for more attention to mental representations. *J. Behav. Therapy Exp. Psychiatry* 67:101510. 10.1016/j.btep.2019.101510 31640848

[B3] Baayen R. H. Schreuder R. (2000). Towards a psycholinguistic computational model for morphological parsing. *Philosop. Trans. R. Soc. Ser. A* 358 1–13.

[B4] Baayen R. H. Wurm L. H. Aycock J. (2007). Lexical dynamics for low-frequency complex words: A regression study across tasks and modalities. *Mental Lexicon* 2 419–463. 10.1075/ml.2.3.06baa 33486653

[B5] Baker M. (2003). *Lexical Categories: Verbs, Nouns, and Adjectives.* Cambridge, MA: Cambridge University Press.

[B6] Beck S. (2011). “Comparison constructions,” in *Semantics: An International Handbook of Nature Language Meaning*, Vol. 2 eds Maienborn C. von Heusinger K. Portner P. (Berlin: Mouton de Gruyter), 1341–1390.

[B7] Berent I. (2013). *The phonological mind.* Cambridge: Cambridge University Press.

[B8] Berent I. Platt M. Theodore R. Balaban E. Fried P. J. Pascual-Leone A. (2020). Speech perception triggers articulatory action: Evidence from mechanical stimulation. *Front. Communicat.* 5:34. 10.3389/fcomm.2020.00034

[B9] Boas H. C. Sag I. A. (2012). *Sign-based construction grammar.* Chicago: CSLI Publications.

[B10] Borer H. (2005a). *In Name Only – Structuring Sense*, Vol. 1 Oxford: Oxford University Press.

[B11] Borer H. (2005b). *The Normal Course of Events – Structuring Sense*, Vol. 2 Oxford: Oxford University Press.

[B100] Borer H. (2013). *Taking Form: Making Sense*, Vol. III. Oxford: Oxford University Press.

[B12] Bowers J. (1993). The syntax of predication. *Linguist. Inq.* 24 591–656.

[B13] Bybee J. L. (2001). *Phonology and language use.* Cambridge: Cambridge University Press.

[B14] Bybee J. L. (2010). *Language, Usage and Cognition.* Cambridge: Cambridge University Press.

[B15] Caha P. (2009). *The Nanosyntax of Case.* Ph D. Thesis, Tromsø: University of Tromsø.

[B16] Caha P. (2018). “Notes on insertion in Distributed Morphology and Nanosyntax,” in *Exploring Nanosyntax*, ed. Baunaz L. De Clercq K. Haegeman L. Lander E. (Oxford: Oxford University Press), 57–88.

[B17] Caha P. Pantcheva M. (2012). “Contiguity Beyond Linearity: Modeling CrossDimensional Syncretisms,” in *Talk presented at Workshop on the Representation and Selection of Exponents* (Norway: University of Tromsø).

[B18] Chater N. Clark A. Goldsmith J. A. Perfors A. (2015). *Empiricism and Language Learnability.* Oxford: Oxford University Press.

[B19] Chomsky N. (1995). *The Minimalist Program.* Cambridge, MA: MIT Press.

[B20] Chomsky N. (2005). Three factors in language design. *Linguist. Inquiry* 36 1–22. 10.1162/0024389052993655

[B21] Chomsky N. (2013). Problems of projection. *Lingua* 130 33–49. 10.1016/j.lingua.2012.12.003

[B22] Christiansen M. H. Chater N. (2016). *Creating language: Integrating evolution, acquisition, and processing.* Cambridge, MA: MIT Press.

[B23] Corver N. (1997). Much-support as a last resort. *Linguist. Inq.* 28 119–164.

[B24] Croft W. A. (2001). *Radical Construction Grammar: Syntactic Theory in Typological Perspective.* Oxford: Oxford University Press.

[B25] Culbertson J. Newport E. L. (2015). Harmonic biases in child learners: In support of language universals. *Cognition* 139 71–82. 10.1016/j.cognition.2015.02.007 25800352 PMC4397919

[B26] Culicover P. W. Jackendoff R. (2005). *Simpler syntax.* Oxford: Oxford University Press.

[B27] de Vaan L. Schreuder R. Baayen R. H. (2007). Regular morphologically complex neologisms leave detectable traces in the mental lexicon. *Mental Lexicon* 2 1–24. 10.1075/ml.2.1.02vaa 33486653

[B28] Embick D. (2015). *The morpheme: a theoretical introduction.* Berlin: Mouton de Gruyter.

[B29] Embick D. Marantz A. (2008). Architecture and blocking. *Linguis. Inquiry* 39 1–53.

[B30] Fábregas A. Putnam M. T. (2020). *Passives and middles in Mainland Scandinavian: Microvariation through exponency.* Berlin: Mouton De Gruyter.

[B31] Fasanella A. Fortuny J. (2016). “Deriving linguistic variation from learnability conditions: The Chunking Procedure,” in *Rethinking Parameters*, eds Eguren L. Fernández-Soriano O. Mendikoetxea A. (Oxford: Oxford University Press), 105–132. 10.1093/acprof:oso/9780190461737.003.0004

[B32] Fedzechkina M. Jaeger T. F. Newport E. L. (2012). Language learners restructure their input to facilitate efficient communication. *Proc. Natl. Acad. Sci.* 109 17897–17902. 10.1073/pnas.1215776109 23071337 PMC3497763

[B33] Fillmore C. Kay P. (1993). *Construction grammar coursebook.* Berkeley: University of California.

[B34] Goldberg A. (1995). *Constructions: a construction grammar approach to argument structure.* Chicago: University of Chicago Press.

[B35] Goldberg A. (2006). *Constructions at work.* Oxford: Oxford University Press.

[B36] Goldberg A. (2019). *Explain me this: Creativity, competition and the partial productivity of constructions.* Princeton, NJ: Princeton University Press.

[B37] Gwilliams L. (2019). How the brain composing morphemes into meaning. *Philosop. Trans. R. Soc. B* 375:20190311. 10.1098/rstb.2019.0311 31840591 PMC6939360

[B38] Hale K. Keyser S. J. (2002). *Prolegomenon to a theory of argument structure.* Cambridge, MA: MIT Press.

[B39] Halle M. Marantz A. (1993). “Distributed Morphology and the pieces of inflection,” in *The view from Building 20*, eds Hale K. Keyser S. J. (Cambridge, MA: MIT Press), 111–176.

[B40] Haugen J. D. Siddiqi D. (2016). “Towards a restricted realization theory: multimorphemic monolistemicity, portmanteaux and post-linearization spanning,” in *Morphological metatheory*, eds Siddiqi D. Harley H. (Amsterdam: John Benjamins), 343–387. 10.1075/la.229.12hau

[B41] Hockett C. F. (1958). *A Course in Modern Linguistics.* New York: MacMillan.

[B42] Hopper P. J. (1998). *Emergent Grammar. In The new psychology of language.* New Jersey: Lawrence Erlbaum, 155–175.

[B43] Jackendoff R. (2017). In defense of theory. *Cognit. Sci.* 41 185–212. 26611772 10.1111/cogs.12324

[B44] Jackendoff R. Audring J. (2020). *The texture of the lexicon: Relational Morphology and the Parallel Architecture.* Oxford: OUP Oxford.

[B45] Kay P. P. Fillmore C. (1999). Grammatical constructions and linguistic generalizations: the ‘what’s X doing Y’ construction.’. *Language* 75 1–33. 10.1353/lan.1999.0033

[B46] Klein E. (1991). “Comparatives,” in *Semantik: eininternationalesHandbuch der zeitgenössischenForschung*, eds von Stechow A. Wunderlich D.. Berlin: Mouton de Gruyter.

[B47] Kuperman V. Bertram R. Baayen R. H. (2010). Processing trade-offs in the reading of Dutch derived words. *J. Memory Language* 62 83–97. 10.1016/j.jml.2009.10.001

[B48] Langacker R. W. (1987). *Foundations of cognitive grammar*, Vol. 1. Stanford: Stanford University Press.

[B49] Langacker R. W. (2000). “A dynamic usage-based model,” in *Usage-based models of language*, eds Barlow M. Kemmer S. (Stanford, CA: CSLI), 1–63.

[B50] Larson R. (2014). *On Shell Structure.* London: Routledge.

[B51] Lightfoot D. (2020). *Born to parse: How children select their languages.* Cambridge, MA: MIT Press.

[B52] Londahl T. (2014). *Phrase structure and argument structure.* Oxford: Oxford University Press.

[B53] Marantz A. (2013). No escape from morphemes in morphological processing. *Lang. Cognit. Proc.* 28 905–916. 10.1080/01690965.2013.779385

[B54] Matthews P. H. (1991). *Morphology.* Cambridge: Cambridge University Press.

[B55] McCauley S. M. Monaghan P. Christiansen M. H. (2015). “Language Emergence in Development,” in *The Handbook of Language Emergence*, eds Whinney B. O’Grady W. (New Jersey: John Wiley & Sons, Inc), 415–436. 10.1002/9781118346136.ch19

[B56] McGinnis M. (2002). On the systematic aspect of idioms. *Linguist. Inq.* 33 665–672. 10.1162/ling.2002.33.4.665

[B57] Meteyard L. Cuadrado S. R. Bahrami B. Vigliocco G. (2012). Coming of age: A review of embodiment and the neuroscience of semantics. *Cortex* 48 788–804. 10.1016/j.cortex.2010.11.002 21163473

[B58] Michaelis L. A. (2012). “Making the case for Construction Grammar,” in *Sign-based Construction Grammar*, eds Boas H. C. Sag I. (Stanford, CA: CSLI Publications), 31–68.

[B59] Moscoso P. M. Fermín, Kostić A. Baayen R. H. (2004). Putting the bits together: An information theoretical perspective on morphological processing. *Cognition* 94 1–18. 10.1016/j.cognition.2003.10.015 15302325

[B60] Pietroski P. (2018). *Conjoining Meanings: Semantics Without Truth Values.* Oxford: Oxford University Press.

[B61] Plag I. Baayen R. H. (2009). Suffix ordering and morphological processing. *Language* 85 109–152. 10.1353/lan.0.0087

[B62] Ramchand G. (2008). *Verb Meaning and the Lexicon: A First-Phase Syntax.* Cambridge: Cambridge University Press.

[B63] Ramchand G. (2018). *Situations and syntactic structures.* Cambridge, MA: MIT Press.

[B64] Ramscar M. Hendrix P. Love B. C. Baayen R. H. (2013). Learning is not decline: The mental lexicon as a window into cognition across the lifespan. *Mental Lexicon* 8 450–481. 10.1075/ml.8.3.08ram 33486653

[B65] Robins R. H. (1959). In defense of WP. *Trans. Philol. Soc.* 58 116–144.

[B66] Schreuder R. Baayen R. H. (1995). “Modeling morphological processing,” in *Morphological aspects of language processing*, ed. Feldman L. B. (New Jersey: Lawrence Erlbaum Associates), 131–154.

[B67] Schwarzschild R. (2008). The semantics of comparatives and other degree constructions. *Lang. Linguist. Compass* 2 308–331. 10.1111/j.1749-818X.2007.00049.x

[B68] Siddiqi D. (2018). “Distributed Morphology,” in *The Oxford Handbook of Morphological Theory*, eds Audring J. Masini F. (Oxford: Oxford University Press), 143–166.

[B69] Spencer A. (1991). *Morphological theory.* Oxford: Blackwell.

[B70] Spencer A. (2013). *Lexical relatedness.* Oxford: Oxford University Press.

[B71] Starke M. (2009). Nanosyntax: A Short Primer to a New Approach to Language. *Nordlyd* 36 1–6.

[B72] Stroik T. Putnam M. (2013). *The structural design of language.* Cambridge: Cambridge University Press.

[B73] Stump G. T. (1998). “Inflection,” in *The Handbook of Morphology*, eds Zwicky A. Spencer A. (London: Wiley), 13–44.

[B74] Stump G. T. (2001). *Inflectional morphology: a theory of paradigm structure.* Cambridge: Cambridge University Press.

[B75] Svenonius P. (2016). “Spans and words,” in *Morphological metatheory. Amsterdam*, eds Siddiqi D. Harley H. (Amsterdam: John Benjamins), 201–223. 10.1075/la.229.07sve

[B76] Taft M. Forster K. I. (1975). Lexical storage and retrieval of prefixed words. *J. Verbal Learn. Verbal Behav.* 14 638–647. 10.1016/S0022-5371(75)80051-X

[B77] Tettamanti M. Moro A. (2012). Can syntax appear in a mirror (system)? 2012. *Cortex* 48 923–935. 10.1016/j.cortex.2011.05.020 21718980

[B78] Tomasello M. (2003). *Constructing a language: A usage-based theory of language acquisition.* Cambridge, MA: Harvard University Press.

[B79] Travis L. (1984). *Parameters and Effects of Word Order Variation.* Ph.D. dissertation, Cambridge, MA: MIT Press.

[B80] Trotzke A. Zwart J. (2014). “The complexity of narrow syntax: minimalism, representational economy, and simplest Merge,” in *Measuring grammatical complexity*, eds Newmeyer F. J. Preston L. B. (Oxford: Oxford University Press), 128–147. 10.1093/acprof:oso/9780199685301.003.0007

[B81] Yang C. (2016). *The Price of Linguistic Productivity: How Children Learn to Break the Rules of Language.* Cambridge, MA: MIT Press.

